# Characterization of Low-Frequency Local Field Potential Activity and Functional Network Organization in the Pigeon Nidopallium Caudolaterale Across Entrance-Related Behavioral States

**DOI:** 10.3390/ani16193013

**Published:** 2026-09-24

**Authors:** Zheng Xu, Deyu Zhao, Zhizhong Wang

**Affiliations:** School of Electrical and Information Engineering, Zhengzhou University, Zhengzhou 450001, China

**Keywords:** pigeon, nidopallium caudolaterale, goal-directed behavior, functional network topology, behavioral state decoding

## Abstract

Goal-directed behavior requires animals to integrate environmental information and select appropriate actions—functions commonly associated with the mammalian prefrontal cortex (PFC). The avian nidopallium caudolaterale (NCL) is functionally analogous to the PFC, but its neural activity across different actions at a decision point remains unclear. At a maze arm entrance, pigeons may enter, hesitate, or leave according to available information. We recorded local field potential (LFP) activity from the NCL in three adult pigeons performing a goal-directed maze task. Direct-enter, hesitation-leave, and no-food-leave were compared with direct-leave by analyzing the theta- and alpha-band power, interchannel coherence, functional network topology, and behavioral state decoding. Compared with direct-leave, the other three behaviors were generally accompanied by higher power and interchannel coherence in both frequency bands. The mean node strength and clustering coefficient also tended to be higher, whereas global efficiency and modularity exhibited more complex patterns. Decoding suggested that power and functional network features contained information relevant to differentiating these behavioral states, with the overall performance being highest for no-food-leave versus direct-leave. These findings indicate that NCL low-frequency neural population activity, interchannel coordination, and functional network organization are associated with entrance-related behavioral states and may contribute to entrance-related information processing and subsequent behavioral adjustment.

## Simple Summary

Understanding how animals use information from their surroundings to decide what to do next can help us to better understand the brain mechanisms that support flexible behavior. In this study, we examined brain activity in three pigeons while they moved through a maze and made different choices at the entrances to maze arms, such as entering, hesitating, looking inside, or leaving. We found that brain activity in a region involved in higher cognitive functions generally changed depending on the pigeons’ behavior. The clearest differences were seen when pigeons looked inside a maze arm, found no food, and then left. These results show that this brain region changes its activity according to different behavioral situations at a decision point. Our study therefore achieved its aim of identifying brain activity patterns associated with different entrance-related behaviors and provides a basis for future research on how birds use environmental information to guide their actions.

## 1. Introduction

Goal-directed behavior is an important capacity that enables animals to adapt to complex environments. When searching for food, evaluating environmental cues, and selecting action paths, animals must integrate visual and spatial information with the task context. They use this information to choose whether to approach, enter, or leave [1]. Characterizing brain activity across behavioral states in a naturalistic maze task can therefore help to clarify the neural basis of action selection and behavioral adjustment during goal-directed behavior.

### 1.1. Avian Nidopallium Caudolaterale (NCL) and the Regulation of Goal-Directed Behavior

Studies in mammals indicate that the prefrontal cortex (PFC) is involved in integrating environmental information, spatial goals, and action-related information. It also plays an important role in goal-directed decision-making. In rat spatial navigation tasks, PFC neurons represent spatial goals and route choices, and their activity is closely associated with animals’ approach to goals [2]. The medial PFC has also been implicated in memory and decision-making. This involvement may enable animals to adjust their action strategies according to the task context [3].

In birds, the NCL is functionally analogous to the mammalian PFC [4,5,6,7]. Lesion and electrophysiological studies further implicate the avian NCL in learning, cognitive flexibility, executive control, and rule-guided decisions [8,9,10]. Previous maze studies have shown that pigeon NCL activity and functional network organization change during goal-directed behavior and target detection [11,12]. These findings suggest that the NCL may integrate task-related information and support action selection and behavioral adjustment during goal-directed behavior.

Previous maze studies of the pigeon NCL have mainly compared neural activity or network features before and after target detection. However, NCL activity across distinct entry and departure states at the maze arm entrance remains insufficiently characterized. After approaching a maze arm entrance, pigeons may display one of four behavioral states: direct-enter, direct-leave, hesitation-leave, or no-food-leave. These states may involve different processes of information acquisition and action selection. Comparing NCL neural activity and functional network features across these states may further clarify the role of the NCL in naturalistic goal-directed behavior.

### 1.2. Local Field Potential (LFP) Activity and NCL Functional Networks

LFP activity primarily reflects aggregate changes in synaptic activity and transmembrane currents within neural populations in the recorded region. Its spectral power can characterize neural population activity across different frequency ranges [13,14,15,16]. Beyond local spectral activity, interchannel coherence quantifies the synchronization of two LFP signals at a specific frequency. It thereby reflects coordinated activity across recording channels [17,18,19]. Representing interchannel coherence as a functional network allows us to further characterize the coordinated organization of neural populations and its relationship with behavioral states at the network level [20,21].

A functional network consists of nodes and the connections between them. In electrophysiological functional networks, recording channels serve as nodes, interchannel coherence represents connection weights, and the mean coherence across all channel pairs reflects overall synchronization. Metrics used to characterize the NCL functional network topology include the mean node strength, clustering coefficient, global efficiency, and modularity [22,23,24,25,26]. The mean node strength reflects the average sum of the connection weights linking each node to all other nodes. The clustering coefficient describes how closely neighboring nodes are interconnected, whereas global efficiency reflects the network’s overall capacity for information integration. Modularity describes the extent to which the network can be divided into relatively independent modules. Together, these metrics characterize the coordinated organization of neural populations within the NCL in terms of connection strength, local clustering, global integration, and modular segregation.

The organization of a functional network is not fixed but changes with task demands and behavioral states. Different states may be accompanied by differences in connection strength, local clustering, global integration, and modular organization. Combining interchannel coherence with the functional network topology can therefore help to characterize state-related NCL network organization from the perspective of coordinated neural population activity [20,26]. Based on this framework, the present study used a goal-directed maze task in which pigeons could exhibit four distinct behavioral responses after approaching a maze arm entrance: direct-enter, direct-leave, hesitation-leave, and no-food-leave. Direct-leave served as the common reference state, and direct-enter, hesitation-leave, and no-food-leave were each compared with it. We analyzed the low-frequency LFP power, interchannel coherence, and NCL functional network topology across these entrance-related behavioral states. We further assessed whether these neural features could support behavioral state decoding.

## 2. Materials and Methods

### 2.1. Animals

Three adult pigeons weighing 400–500 g were used and were designated as P092, P106, and P653. The pigeons were obtained from a commercial breeding farm. However, the specific breed or genetic strain was not documented by the supplier. Each pigeon was housed individually in a pigeon cage under a 12 h light/12 h dark cycle. Water was available ad libitum throughout the experiment, and food intake was restricted to maintain body weight at 80–85% of the free-feeding weight. All procedures complied with the requirements for the care and use of experimental animals under the 2006 Animal Law (China). All procedures were approved by the Life Science Ethical Review Committee of Zhengzhou University.

### 2.2. Behavioral Apparatus and Task Procedure

As shown in Figure 1a, the apparatus was an open-top square maze constructed from polyvinyl chloride (PVC) panels. It measured 1.6 m long, 1.6 m wide, and 0.6 m high. A central obstacle measuring 20 cm long, 20 cm wide, and 60 cm high created four surrounding maze arms, designated A, B, C, and D. Each maze arm entrance was 20 cm wide.

During the experiment, a target object containing 6 g of food was randomly placed at the end of one maze arm, while the other three maze arms contained no food. The food reward location was changed randomly according to the experimental procedure. A photoelectric sensor was installed at each maze arm entrance to record the time at which the pigeon triggered the sensor. A barrier structure used to secure the sensor was also installed at each maze arm entrance. This structure both defined the sensor-triggering position and blocked a direct line of sight from outside the maze arm entrance to inside the maze arm. Thus, while remaining outside the maze arm entrance, pigeons could not determine whether food was present by merely extending their heads. They had to move further past the maze arm entrance to confirm its presence or absence.

During the experiment, pigeons moved freely within the maze and approached different maze arm entrances. After approaching a maze arm entrance, a pigeon could enter the maze arm, remain near the maze arm entrance to observe, or leave directly. After completing one entrance-related behavior, the pigeon continued moving through the maze and could approach another maze arm entrance. The behavioral-state classification procedure is shown in Figure 1b.

Before the formal experiment, pigeons underwent preoperative and postoperative training. The training criterion was met only when a pigeon completed at least 10 target searches and obtained food rewards within 30 min on each of four consecutive training days. Preoperative training comprised nine training days separated by 1 d intervals and spanned 18 d. After surgery, pigeons rested for 7–10 d without training. After recovery, they underwent five postoperative training days, again separated by 1 d intervals and spanning 9 d. Pigeons then rested for 1 d before each participated in six formal experimental days separated by 1 d intervals and spanning 11 d. Electrophysiological signals were not collected during either the preoperative or postoperative training period.

During the formal experiment, photoelectric sensors and an overhead camera (X5-1080p, S-YUE ShengYue, Yueqing City, China) recorded pigeon behavior synchronously. Sensor timestamps and video recordings were subsequently combined to identify and classify the behavioral state for each entrance-related trial. A trial was excluded from subsequent analyses if its video recording was incomplete, its sensor timestamp was anomalous, or the behavioral state could not be classified reliably.

### 2.3. Surgery and Electrophysiological Recording

Before surgery, pigeons were anesthetized with 3% sodium pentobarbital (0.2 mL/100 g body weight). This pentobarbital-based anesthetic protocol was adopted following established procedures used in previous pigeon neurophysiological studies involving electrode implantation [27]. After anesthesia, each pigeon was secured in a custom stereotaxic apparatus. Using the stereotaxic atlas of the pigeon brain [28], a 16-channel microneedle array electrode (4 × 4 array, KD-MEA-16, KedouBC, Suzhou, China) was implanted in the left nidopallium caudolaterale (NCL). The arrangement of the recording channels is shown in Figure 1c. The implantation coordinates for P092 were mediolateral (ML) 7.5 mm, anteroposterior (AP) 5.5 mm, and dorsoventral (DV) 3.5 mm. The coordinates for both P106 and P653 were ML 7.5 mm, AP 5.05 mm, and DV 3.75 mm.

During the formal experiment, neural signals from 16 recording channels were acquired using a Hermes Wireless Data Acquisition and Recording System (Jiangsu Yige Biotechnology Co., Ltd., Nanjing, China). The system amplified, filtered, and digitized the neural signals and stored them on a memory card at a sampling rate of 30 kHz. Neural signals, photoelectric sensor timestamps, and behavioral videos were recorded synchronously for the subsequent alignment of the electrophysiological signals with entrance-related behavioral events. Some of the electrophysiological recordings analyzed in the present study were obtained within the experimental framework previously described by Wang et al. [12]; the present study addresses a distinct research question by classifying entrance-related behavioral states and applying analyses tailored to these states.

Sodium pentobarbital was not administered during the formal electrophysiological recording sessions. Formal recordings were conducted only after a 7–10 d postoperative recovery period and subsequent behavioral training.

### 2.4. Histological Verification and Confirmation of Electrode Locations

After all experimental data had been collected, pigeons were anesthetized with 3% sodium pentobarbital (0.2 mL/100 g body weight). A continuous 30 s negative direct-current pulse was then delivered through the implanted electrode to create an electrolytic lesion at the implantation site. For the pigeon represented in Figure 1c, the current used was 0.30 mA. The pigeons were subsequently perfused transcardially with physiological saline followed by 4% paraformaldehyde fixative.

After perfusion, the brains were removed and post-fixed in 4% paraformaldehyde for 1 d. They were then placed sequentially in 15% sucrose solution for 1 d and 30% sucrose solution for 3 d for graded cryoprotection. After cryoprotection, the brain tissue was embedded for cryosectioning and cut into 40 μm sections using a Leica cryostat. The sections were dried in a constant-temperature oven at 37 °C for 12 h and then subjected to Nissl staining according to standard histological procedures.

Stained brain sections were scanned using a VS200 research-grade whole-slide scanning system. The electrolytic lesions and their anatomical locations were examined using the OlyVIA software (V4.1) to confirm that the recording sites were located within the NCL. A representative histological section used for recording-site verification is shown in Figure 1c.

### 2.5. Behavioral State Classification

An entrance-related trial was defined as one behavioral response occurring after a pigeon approached a maze arm entrance. Behavioral states were classified from synchronized overhead video recordings and photoelectric sensor timestamps according to predefined operational criteria. The classification followed a sequential decision procedure based on whether the pigeon entered the maze arm, displayed obvious hesitation near the entrance, crossed the entrance barrier sufficiently to obtain visual access to the maze arm interior, and subsequently entered or left the arm.

Direct-enter was assigned when the pigeon continued directly into the maze arm after approaching the entrance without an obvious pause or hesitation. Direct-leave was assigned when the pigeon turned away and left the entrance without entering the maze arm or displaying obvious hesitation. Hesitation-leave was assigned when the pigeon slowed, paused, looked around, or extended its head near the entrance and subsequently left, while its eyes remained outside the entrance barrier and it therefore had no direct visual access to the interior of the maze arm. No-food-leave was assigned when the pigeon moved sufficiently far for its eyes to pass the entrance barrier, thereby obtaining visual access to the maze arm interior, and subsequently left after observing that no food was present.

Thus, two principal decision boundaries were used in the classification. The presence or absence of obvious hesitation distinguished direct-leave from hesitation-leave, whereas whether the pigeon’s eyes passed the entrance barrier and obtained visual access to the maze arm interior distinguished hesitation-leave from no-food-leave. Photoelectric sensor timestamps were used as supplementary temporal information but were not used alone to distinguish these behavioral states.

To assess the reliability of the behavioral classification, a second observer, blinded to the original behavioral labels and electrophysiological results, independently classified all entrance-related trials included in the analyses according to the same predefined operational criteria. Interobserver reliability was quantified using the percentage of identical classifications and Cohen’s kappa coefficient.

Additional descriptive analyses quantified the occurrence proportion of each behavioral state, the behavioral response duration from approach to the maze arm entrance to completion of the corresponding response, and the distribution of the four behavioral states across the formal recording sessions.

### 2.6. Neural Signal Preprocessing and Analysis Time Windows

Raw electrophysiological signals from the 16 recording channels were band-pass-filtered at 1–250 Hz. The 50 Hz line noise and its harmonics at 100 and 150 Hz were removed, after which the signals were downsampled to 1 kHz. For all neural signals, the time at which the pigeon approached the maze arm entrance was defined as 0 s. Event-locked signal segments from −1 to 4 s were then extracted. The analysis windows for the four behavioral states are illustrated in Figure 2a. The analyses focused on the theta band (4–8 Hz) and alpha band (8–12 Hz) for power, coherence, and functional network analyses. Representative LFP signals and their components in the theta (4–8 Hz) and alpha (8–12 Hz) bands are shown in Figure 2b.

For direct-enter and direct-leave, the 0–2 s window was analyzed for both behaviors. For hesitation-leave and direct-leave, the 0–2 s window was analyzed for both behaviors. For no-food-leave, the 1.5–3.5 s window was analyzed and compared with the 0–2 s window for direct-leave. The delayed window for no-food-leave was used because the pigeon had to move farther past the barrier after approaching the maze arm entrance. Only then could it observe whether food was present inside the maze arm.

### 2.7. Power and Multitaper Coherence Analyses

For each entrance-related trial, the power in the theta band (4–8 Hz) and alpha band (8–12 Hz) was calculated separately within the corresponding analysis window using the short-time Fourier transform. The transform used a window length of 512 samples and a step size of 10 samples. The power was averaged within each frequency band and then $\log_{10}$ transformed. At each time point, the median across the 16 recording channels was calculated, followed by averaging over the target time window to obtain the MeanLogPower:

(1) $$\mathrm{MeanLogPower}={\mathrm{mean}}_{t}\left[{\mathrm{median}}_{c}\left(\log_{10}P_{c}\left(t\right)\right)\right]$$

where $P_{c}\left(t\right)$ denotes the mean power of recording channel *c* in the corresponding frequency band at time *t*.

Interchannel coherence was calculated using the multitaper method (MTM) [29,30]. This method applies multiple mutually orthogonal discrete prolate spheroidal sequences (DPSS) to the same data segment. This reduces spectral leakage and spectral estimation variance, thereby improving the stability of coherence estimates from short data segments. The MTM time-bandwidth product was set to NW=3, and the number of tapers was set to K=5. For each trial, the interchannel coherence in the theta and alpha bands was calculated over the entire analysis window assigned to its behavioral state. For recording channels *x* and *y*, the coherence at frequency *f* was defined as

(2) $$C_{xy}\left(f\right)=\frac{{\left|\frac{1}{K}\sum_{k=1}^{K}X_{k}\left(f\right)Y_{k}^{*}\left(f\right)\right|}^{2}}{\left(\frac{1}{K}\sum_{k=1}^{K}{\left|X_{k}\left(f\right)\right|}^{2}\right)\left(\frac{1}{K}\sum_{k=1}^{K}{\left|Y_{k}\left(f\right)\right|}^{2}\right)}$$

where $X_{k}\left(f\right)$ and $Y_{k}\left(f\right)$ denote the Fourier transforms of the signals from the two recording channels after the application of taper *k*, respectively, and $Y_{k}^{*}\left(f\right)$ denotes the complex conjugate. Coherence values were averaged separately across the theta and alpha bands to construct a 16×16 coherence matrix for each trial. The matrix diagonal was excluded, and the mean of the off-diagonal upper-triangular elements was used as the mean coherence for that trial.

### 2.8. Functional Network Construction and Network Metrics

NCL functional networks were constructed from the 16×16 MTM coherence matrices for the theta and alpha bands in each trial. The network nodes represented the 16 recording channels, and the coherence value $C_{ij}$ between two nodes represented their functional connection weight. The diagonal of the coherence matrix was excluded from network construction. The overall procedure from coherence matrix generation to weighted functional network construction is illustrated in Figure 2c.

To control for network sparsity, off-diagonal connections in the coherence matrix were ranked in descending order of coherence, and the strongest 20%, 25%, and 30% of connections were retained. A density of 25% was used for the primary analysis, while 20% and 30% were used to examine the stability of the results across network density settings, because graph metrics can depend on the connectivity density [31]. The retained connections formed a binary adjacency matrix *B*, in which retained connections were assigned a value of 1 and all other connections had a value of 0. This matrix represented the binary network. The original coherence matrix was then multiplied elementwise by the binary adjacency matrix to obtain a weighted adjacency matrix *W*, which represented the weighted network:

(3) $$W_{ij}=C_{ij}B_{ij}$$

The mean node strength and modularity were calculated from the weighted network, whereas the clustering coefficient and global efficiency were calculated from the binary network. The strength of node *i* was the sum of the weights of all retained connections between that node and the other nodes [22]. The mean node strength was defined as

(4) $$s_{i}=\sum_{j\neq i}W_{ij},\;\bar{s}=\frac{1}{N}\sum_{i=1}^{N}s_{i}$$

where $s_{i}$ denotes the strength of node *i*, and *N* denotes the number of network nodes.

The clustering coefficient describes the extent to which the neighbors of a node are interconnected [23,26]. The clustering coefficient of node *i* and its network mean were defined as

(5) $$C_{i}^{\mathrm{clu}}=\frac{2T_{i}}{k_{i}\left(k_{i}-1\right)},\;{\bar{C}}^{\mathrm{clu}}=\frac{1}{N}\sum_{i=1}^{N}C_{i}^{\mathrm{clu}}$$

where $T_{i}$ denotes the number of connections present among the neighbors of node *i*, and $k_{i}$ denotes the degree of node *i*. When $k_{i}<2$, $C_{i}^{\mathrm{clu}}=0$.

Global efficiency was used to measure the efficiency of information transfer between different nodes in the network and was defined as [24,26]

(6) $$E_{\mathrm{glob}}=\frac{1}{N(N-1)}\sum_{i\neq j}\frac{1}{d_{ij}}$$

where $d_{ij}$ denotes the shortest-path length between nodes *i* and *j* in the binary network. If no path existed between two nodes, their efficiency was assigned a value of 0.

Modularity was used to quantify the extent to which the network could be partitioned into distinct functional modules [25]. A greedy module-merging method was used to determine the module partition [32], and the weighted modularity was defined as

(7) $$Q=\frac{1}{2m}\sum_{i,j}\left(W_{ij}-\frac{s_{i}s_{j}}{2m}\right)\delta \left(g_{i},g_{j}\right)$$

where $m=\frac{1}{2}\sum_{i,j}W_{ij}$ denotes the total connection weight of the network, and $g_{i}$ and $g_{j}$ denote the modules to which the nodes belong. When two nodes belonged to the same module, $\delta (g_{i},g_{j})=1$; otherwise, it was 0.

### 2.9. Decoding Analysis

To assess whether NCL neural activity features could be used to distinguish between entrance-related behavioral states, the trials from the three pigeons were pooled. Separate binary classification models were constructed for direct-enter versus direct-leave, hesitation-leave versus direct-leave, and no-food-leave versus direct-leave. Candidate features included the MeanLogPower in the theta and alpha bands. At the 25% network density, they also included the mean node strength, clustering coefficient, global efficiency, and modularity in both bands. This yielded 10 features in total.

The classifiers were a linear support vector machine (linear SVM) and elastic-net logistic regression [33,34]. Feature standardization, feature selection, and model-parameter optimization were performed using only the training data and were determined by internal cross-validation within the training set [35,36]. Class-balanced weights were used during model training to reduce the effects of differences in trial counts between classes.

Leave-one-session-out (LOSO) cross-validation was first performed. In each iteration, one complete experimental session served as the test set, while all remaining sessions served as the training set. This procedure was used to evaluate model generalization to an unseen session within the pooled dataset. As a supplementary analysis, stratified 10-fold cross-validation was repeated 50 times on the pooled trials from the three pigeons. Model performance was evaluated using accuracy and the area under the receiver operating characteristic curve (AUC) [37]. For the LOSO results, 500 within-session label permutations were additionally performed to test whether the AUC was significantly higher than chance [38].

### 2.10. Statistical Analysis

The power, mean coherence, and network metrics were analyzed separately for P092, P106, and P653, with each entrance-related trial treated as the statistical unit. Differences between direct-enter and direct-leave, hesitation-leave and direct-leave, and no-food-leave and direct-leave were assessed using two-sided Wilcoxon rank-sum tests.

To control for false-positive findings arising from multiple comparisons, raw *p* values were adjusted for the false discovery rate (FDR) using the Benjamini–Hochberg procedure [39]. Within each pigeon, FDR correction was performed separately for the power, coherence, and network metric analyses. For power and coherence, each correction family included the three behavioral comparisons and two frequency bands. For network metrics, each correction family included all behavioral comparisons, frequency bands, network densities, and network metrics. For the decoding analysis, the six AUC permutation-test *p* values were treated as a single correction family. These values were obtained from the two classifiers across the three behavioral comparisons and were adjusted using the Benjamini–Hochberg procedure. A raw p<0.05 indicated uncorrected nominal significance, whereas an FDR-adjusted q<0.05 indicated statistical significance after correction. Significance markers in the figures are based on the raw *p* values, with “*” indicating p<0.05, “**” indicating p<0.01, and “***” indicating p<0.001. FDR-adjusted *q* values are reported separately in the main text or tables. All statistical analyses were performed in MATLAB R2022b (MathWorks, Natick, MA, USA).

## 3. Results

### 3.1. Entrance-Related Trials Included in the Analysis

For P092, P106, and P653, 60 entrance-related trials were included for each of direct-enter, direct-leave, hesitation-leave, and no-food-leave, yielding 240 entrance-related trials per pigeon. When each pigeon was analyzed separately, both groups in each of the three behavioral comparisons contained 60 entrance-related trials. These trials were used in the subsequent power, mean coherence, functional network, and behavioral state decoding analyses.

The interobserver assessment showed the high reliability of the behavioral classification, with 98.19% agreement and a Cohen’s κ of 0.976. Descriptive analyses based on all classified entrance-related behavioral events from the formal experiments showed that hesitation-leave had the highest occurrence proportion in all three pigeons, while direct-leave showed the shortest median behavioral response duration and no-food-leave showed the longest. All four behavioral states were distributed throughout the formal recording sessions, without obvious concentration in a particular session.

### 3.2. Power Differences in the Theta and Alpha Bands Across Entrance-Related Behavioral States

The distributions of the MeanLogPower in the theta and alpha bands across entrance-related behavioral states are shown in Figure 3, and descriptive statistics are provided in Table 1. Throughout this section, “significant” refers to q<0.05 after false discovery rate (FDR) correction. Results marked with asterisks in the figures are based only on raw *p* values, whereas the main text reports FDR-adjusted *q* values. All median differences below were calculated as the corresponding behavioral state minus direct-leave.

In the theta band, the MeanLogPower was significantly higher for direct-enter than for direct-leave in P092 and P653. P106 also showed a numerical increase, although the difference was not significant (P092: median difference = 0.241, $q=9.82\times 10^{-4}$; P106: median difference = 0.206, q=0.6064; P653: median difference = 0.273, q=0.0386; Figure 3a). Thus, the direction of change was consistent across pigeons, but statistical significance differed among pigeons.

For hesitation-leave versus direct-leave, the MeanLogPower in the theta band was numerically higher in all three pigeons, but the increase was significant only in P092 (P092: median difference = 0.196, q=0.0018; P106: median difference = 0.025, q=0.6064; P653: median difference = 0.053, q=0.5545; Figure 3c).

For no-food-leave versus direct-leave, the MeanLogPower in the theta band was significantly higher in P092 and P653. In P106, the value was essentially unchanged, with a very slight numerical decrease (P092: median difference = 0.414, $q=7.30\times 10^{-5}$; P106: median difference = −0.001, q=0.9551; P653: median difference = 0.202, q=0.0259; Figure 3e). In P092, this comparison had the largest median difference among the three comparisons in the theta band.

In the alpha band, the MeanLogPower was significantly higher for direct-enter than for direct-leave in P092 and P653. P106 also showed a numerical increase, although the difference was not significant (P092: median difference = 0.206, q=0.0021; P106: median difference = 0.094, q=0.6064; P653: median difference = 0.446, q=0.0345; Figure 3b). As in the theta band, the direction of change was consistent across pigeons, but statistical significance differed among pigeons.

For hesitation-leave versus direct-leave, the MeanLogPower in the alpha band was numerically higher in all three pigeons, but the increase was significant only in P092 (P092: median difference = 0.231, $q=1.15\times 10^{-4}$; P106: median difference = 0.038, q=0.6064; P653: median difference = 0.207, q=0.0792; Figure 3d).

For no-food-leave versus direct-leave, the MeanLogPower in the alpha band was significantly higher in P092 and P653. P106 also showed a numerical increase, although the difference was not significant (P092: median difference = 0.413, $q=7.30\times 10^{-5}$; P106: median difference = 0.127, q=0.6064; P653: median difference = 0.482, q=0.0018; Figure 3f). In P092, this comparison had the largest median difference among the three comparisons in the alpha band.

Overall, the power changes showed a high degree of directional consistency across the three pigeons. The MeanLogPower was higher in all behavioral comparisons except no-food-leave versus direct-leave in the theta band for P106, which was essentially unchanged with a slight decrease. The magnitude and statistical significance of the differences varied among pigeons.

### 3.3. Mean Coherence Differences in the Theta and Alpha Bands Across Entrance-Related Behavioral States

The mean coherence in the theta and alpha bands across entrance-related behavioral states is shown in Figure 4.

In the theta band, P092 showed significantly higher mean coherence for direct-enter, hesitation-leave, and no-food-leave than for direct-leave (direct-enter versus direct-leave: $q=8.66\times 10^{-6}$; hesitation-leave versus direct-leave: $q=1.43\times 10^{-5}$; no-food-leave versus direct-leave: $q=8.66\times 10^{-6}$; Figure 4a,c,e). The difference was largest for no-food-leave versus direct-leave.

P106 showed a slight numerical increase for direct-enter versus direct-leave and slight numerical decreases for hesitation-leave and no-food-leave versus direct-leave. None of the three differences reached statistical significance (all q=0.9182). In P653, the mean coherence was significantly higher for no-food-leave than for direct-leave (q=0.0094). It was numerically higher for hesitation-leave than for direct-leave, although the difference was not significant (q=0.4720). The mean coherence was similar between direct-enter and direct-leave, with a slight numerical decrease that was not significant (q=0.9699). Thus, in the theta band, only the no-food-leave versus direct-leave comparison was significant in two pigeons. The directions and statistical significance of the other comparisons differed among pigeons.

In the alpha band, P092 likewise showed significantly higher mean coherence for direct-enter, hesitation-leave, and no-food-leave than for direct-leave (direct-enter versus direct-leave: $q=1.12\times 10^{-6}$; hesitation-leave versus direct-leave: $q=8.22\times 10^{-8}$; no-food-leave versus direct-leave: $q=1.12\times 10^{-6}$; Figure 4b,d,f). The difference was largest for no-food-leave versus direct-leave.

P106 showed slight numerical increases for direct-enter and hesitation-leave versus direct-leave and a slight numerical decrease for no-food-leave versus direct-leave. None of the three differences reached statistical significance (all q=0.9182). In P653, the mean coherence was significantly higher for no-food-leave than for direct-leave (q=0.0094). It was also numerically higher for direct-enter and hesitation-leave than for direct-leave, although neither difference was significant (q=0.4720 and q=0.1755, respectively). Thus, in the alpha band, the no-food-leave versus direct-leave comparison was again significant in two pigeons, whereas the other comparisons differed among pigeons.

In the equally weighted mean difference matrix across the three pigeons for hesitation-leave minus direct-leave, most pairs of recording channels had positive differences in both the theta and alpha bands. The distribution of positive changes was more consistent in the alpha band (Figure 4g,h). This matrix showed only the direction and spatial distribution of coherence changes; it did not indicate that every pair of recording channels was statistically significant.

Overall, the mean coherence was numerically higher in most entrance-related behavioral state comparisons, but the directional consistency across pigeons was lower than that observed for the power. In P092, all three behavioral comparisons showed significant increases in both frequency bands. P653 showed a significant increase only for no-food-leave versus direct-leave. None of the comparisons reached statistical significance in P106, and some showed slight numerical decreases. Thus, the magnitude and statistical significance of the differences varied among pigeons, and some comparisons also differed in direction.

### 3.4. Topological Characteristics of NCL Functional Networks Across Entrance-Related Behavioral States

The mean node strength, clustering coefficient, global efficiency, and modularity were further compared at the primary network density of 25%, as shown in Figure 5. Each metric was expressed as the median difference calculated as the corresponding behavioral state minus direct-leave. A positive value indicated a higher value for the corresponding behavioral state, whereas a negative value indicated a higher value for direct-leave. Network densities of 20% and 30% were used for stability analyses.

The mean node strength was numerically higher in most comparisons. In P092, it was significantly higher in both the theta and alpha bands for all three behavioral comparisons (direct-enter versus direct-leave: theta $q=1.97\times 10^{-5}$, alpha $q=7.33\times 10^{-6}$; hesitation-leave versus direct-leave: theta $q=1.73\times 10^{-5}$, alpha $q=6.92\times 10^{-7}$; no-food-leave versus direct-leave: theta $q=1.73\times 10^{-5}$, alpha $q=3.63\times 10^{-6}$). P106 showed numerical increases in the first two comparisons and a numerical decrease for no-food-leave versus direct-leave; none of these differences reached statistical significance. P653 showed numerical increases in all three comparisons, with significant increases in both frequency bands for no-food-leave versus direct-leave (theta q=0.0182, alpha q=0.0201; Figure 5a,b).

The clustering coefficient was likewise numerically higher in most comparisons. P092 showed numerical increases in both frequency bands for all three behavioral comparisons. It was significantly higher in both frequency bands for direct-enter versus direct-leave (theta q=0.00375, alpha q=0.00737). It was significantly higher only in the alpha band for hesitation-leave versus direct-leave ($q=1.84\times 10^{-4}$). For no-food-leave versus direct-leave, it was significantly higher in both frequency bands (theta $q=2.80\times 10^{-4}$, alpha $q=6.93\times 10^{-5}$).

P106 showed numerical increases in all comparisons except direct-enter versus direct-leave in the alpha band, which showed a slight numerical decrease. None of these differences reached statistical significance. P653 showed numerical decreases in the theta band for direct-enter and no-food-leave versus direct-leave and numerical increases in the remaining comparisons. None of these differences reached statistical significance (Figure 5c,d).

The direction of change for global efficiency differed markedly among pigeons. In P092, the global efficiency was significantly lower in the theta and alpha bands for all three behavioral comparisons (direct-enter versus direct-leave: theta q=0.00295, alpha $q=5.72\times 10^{-4}$; hesitation-leave versus direct-leave: theta q=0.00409, alpha $q\;=\;1.01\;\times \;10^{-5}$; no-food-leave versus direct-leave: theta q=0.0347, alpha $q=3.53\times 10^{-5}$). P106 showed slight numerical increases in the theta band for direct-enter and no-food-leave versus direct-leave and numerical decreases in the remaining comparisons. None of these differences reached statistical significance. P653 showed numerical increases in both frequency bands for all three behavioral comparisons, but none reached statistical significance (Figure 5e,f).

Modularity showed a mixture of increases and decreases across the comparisons in all three pigeons. P092 showed numerical decreases in both frequency bands for direct-enter and hesitation-leave versus direct-leave and numerical increases in both bands for no-food-leave versus direct-leave. None of these differences reached statistical significance. P106 showed numerical increases in the theta band for direct-enter versus direct-leave, in both bands for hesitation-leave versus direct-leave, and in the theta band for no-food-leave versus direct-leave. The remaining results showed numerical decreases, and none of the differences reached statistical significance. P653 showed numerical increases in both frequency bands for the first two comparisons and numerical decreases in both bands for no-food-leave versus direct-leave. The decrease in the theta band was significant (q=0.0082; Figure 5g,h).

The stability analyses showed that all significant results at the 25% network density retained the same direction and remained significant at the 20% and 30% network densities. Results that reached statistical significance at only one supplementary network density were not treated as primary findings.

Overall, the mean node strength and clustering coefficient were numerically higher in most behavioral comparisons and frequency bands. In contrast, global efficiency and modularity varied more markedly in both magnitude and direction across pigeons.

### 3.5. Behavioral State Decoding Based on Power and Functional Network Features

To test whether NCL neural activity features could be used to distinguish between different entrance-related behavioral states, the trials from P092, P106, and P653 were pooled. The feature set comprised the MeanLogPower in the theta and alpha bands and the mean node strength, clustering coefficient, global efficiency, and modularity in both bands at the 25% network density, yielding 10 dimensions. A linear support vector machine (linear SVM) was used as the primary classifier, and elastic-net logistic regression was used as a supplementary classifier. Model performance was evaluated using accuracy and the area under the receiver operating characteristic curve (AUC). Accuracy was the proportion of test trials classified correctly. The AUC reflected the ability of a model to distinguish the two behavioral states across classification thresholds. Values closer to 1 indicated better discrimination, whereas 0.5 represented chance-level discrimination.

Leave-one-session-out (LOSO) cross-validation was used as the primary validation method. In each iteration, one complete experimental session served as the test set, and the remaining sessions served as the training set. This procedure evaluated model generalization to an unseen session. Because the training and test sessions were fully separated, this method provided a stricter assessment of unseen-session generalization than cross-validation based on the random partitioning of pooled trials. The held-out unit was one experimental session, not one pigeon.

The LOSO cross-validation results showed that the accuracy was generally highest for no-food-leave versus direct-leave and the lowest for hesitation-leave versus direct-leave. For the linear SVM, the accuracy was 69.8%, 61.6%, and 75.3% for direct-enter versus direct-leave, hesitation-leave versus direct-leave, and no-food-leave versus direct-leave, respectively. For elastic-net logistic regression, the corresponding accuracies were 72.9%, 63.0%, and 72.2%. Elastic-net logistic regression had slightly higher accuracy for direct-enter versus direct-leave and hesitation-leave versus direct-leave. The linear SVM had higher accuracy for no-food-leave versus direct-leave (Figure 6a).

The AUC results showed the same ordering across the behavioral comparisons. For the linear SVM, the AUC values were 0.747, 0.691, and 0.795 for direct-enter versus direct-leave, hesitation-leave versus direct-leave, and no-food-leave versus direct-leave, respectively. For elastic-net logistic regression, the corresponding AUC values were 0.777, 0.704, and 0.833. Both models achieved the highest AUC for no-food-leave versus direct-leave and the lowest AUC for hesitation-leave versus direct-leave (Figure 6b–d). After the FDR correction of the six *p* values obtained from the AUC permutation tests, only elastic-net logistic regression for hesitation-leave versus direct-leave reached statistical significance (q=0.0479). None of the other results reached statistical significance after correction. The asterisks in the figure indicate significance based on raw *p* values obtained from 500 within-session label permutations.

As a supplementary analysis, 50 repetitions of stratified 10-fold cross-validation were performed on the pooled trials from the three pigeons. This analysis primarily evaluated classification stability at the pooled-trial level and was not equivalent to a generalization test on unseen sessions or unseen pigeons. Its accuracy results showed the same overall ordering as the LOSO results. For the linear SVM, the accuracy was 68.5% ± 2.2%, 63.8% ± 2.1%, and 81.3% ± 2.4% for direct-enter versus direct-leave, hesitation-leave versus direct-leave, and no-food-leave versus direct-leave, respectively. For elastic-net logistic regression, the corresponding accuracies were 69.7% ± 1.6%, 65.7% ± 1.9%, and 79.6% ± 2.3% (Figure 7a).

For the linear SVM, the three AUC values were 0.753 ± 0.013, 0.710 ± 0.016, and 0.854 ± 0.012. For elastic-net logistic regression, the corresponding values were 0.747 ± 0.023, 0.725 ± 0.016, and 0.846 ± 0.014 (Figure 7b–d). Both models achieved their highest accuracy and AUC for no-food-leave versus direct-leave, whereas the decoding performance was lower for hesitation-leave versus direct-leave.

Overall, with LOSO cross-validation as the primary validation method, the two classifiers achieved different levels of decoding performance across the three behavioral comparisons. The accuracy and AUC were highest for no-food-leave versus direct-leave, followed by direct-enter versus direct-leave, and were lower for hesitation-leave versus direct-leave. Repeated stratified 10-fold cross-validation produced the same performance ordering and provided supplementary trial-level support for these results.

## 4. Discussion

### 4.1. Main Findings

This study used an experimental paradigm focused on entrance-related behavior at the maze arm entrance. It compared LFP activity in the NCL during direct-enter, hesitation-leave, and no-food-leave with that during direct-leave. The analyses covered the power in the theta and alpha bands, interchannel coherence, the functional network topology, and behavioral state decoding. Compared with direct-leave, the low-frequency power and mean coherence were generally higher in the other three entrance-related behavioral states. The mean node strength and clustering coefficient were also higher in most comparisons, whereas global efficiency and modularity showed more complex patterns. Decoding based on power and functional network features further indicated some neural-feature separability among entrance-related behavioral states. The decoding performance was numerically highest for no-food-leave versus direct-leave. Together, these findings indicated corresponding changes in NCL low-frequency neural population activity, coordination across recording channels, and functional network organization. These changes occurred when pigeons engaged in information acquisition at the maze arm entrance and subsequently entered or left the maze arm. The findings suggest that the NCL may be involved in processing information at the entrance and subsequent behavioral adjustment.

### 4.2. Theta- and Alpha-Band Power and Interchannel Coherence

Regarding low-frequency power, the other three entrance-related behaviors were generally accompanied by higher power in the theta and alpha bands in the NCL than direct-leave. These patterns suggest the greater engagement of low-frequency neural population activity in the NCL when pigeons continued to approach, paused to evaluate, or looked inside the maze arm. The LFP power primarily reflects aggregate field activity generated by synaptic activity and transmembrane currents across a neural population. It is not equivalent to changes in the firing rates of individual neurons [14,15]. Thus, the observed power changes may reflect adjustments in NCL low-frequency activity when pigeons engaged in further information processing and behavioral organization instead of leaving the entrance directly.

Although the three behaviors differed overtly, each involved a longer or deeper interaction with the maze arm entrance than in direct-leave. Direct-enter involved a continued approach, hesitation-leave included pausing and behavioral adjustment before departure, and no-food-leave added visual sampling inside the maze arm. The more pronounced power changes during no-food-leave may be related to information acquisition from inside the maze arm and subsequent behavioral adjustment. Previous studies have reported task-dependent oscillatory signatures in the avian NCL. Gamma activity increased during goal-directed behavior in pigeons [40], whereas beta and narrow-band gamma activity carried working-memory information in crows [41]. In pigeons, spatial path adjustment was also accompanied by changes in low-frequency power and elevated gamma-band NCL connectivity [42]. Recent anatomical tracing evidence further demonstrates extensive input and output connections of the avian NCL with sensory, associative, and motor-related regions, supporting its role within a distributed forebrain network [43]. Functional evidence in pigeons further indicates increased theta-band hippocampus–NCL synchronization during visual–spatial associative learning [44]. Together, these findings place the entrance-related NCL activity observed here within a broader forebrain framework involving sensory, spatial, and action-related information processing. Future studies using simultaneous recordings from the NCL and interconnected pallial regions may further clarify the circuit-level dynamics underlying these behavioral states.

Meanwhile, the overall pattern of higher mean coherence in the theta and alpha bands indicated stronger rhythmic coordination across NCL recording channels. This stronger coordination accompanied more complex approach, observation, and behavioral adjustment at the entrance. Thus, the joint changes in power and coherence suggest that NCL representations of entrance-related behavior may involve adjustments in both low-frequency neural population activity and interchannel synchronization. Higher coherence indicates the greater coordination of low-frequency activity across NCL recording channels. This pattern may provide complementary evidence for the coordinated involvement of local NCL neural populations in entrance-related behavior.

### 4.3. Changes in NCL Functional Network Topology

Regarding the functional network topology, the mean node strength and clustering coefficient tended to be higher overall. This pattern may reflect stronger functional coupling among local NCL recording channels when pigeons entered, hesitated, or looked inside the maze arm. Together with the changes in power and mean coherence, this result indicates that entrance-related behavior may also involve adjustments in local functional network organization. Stronger local functional connectivity and clustering could facilitate the coordinated processing of entrance-related information by relevant neural populations and contribute to subsequent behavioral organization.

By contrast, global efficiency and modularity did not show a consistent direction of change. Thus, stronger local connectivity was not necessarily accompanied by corresponding changes in overall NCL network integration or modular structure. This pattern suggests that entrance-related behaviors may influence NCL networks mainly through flexible adjustments in local coordination, rather than uniform global network reorganization. This pattern of prominent local changes and inconsistent global changes further suggests that NCL functional networks may support information processing and behavioral transitions through multiple organizational configurations.

### 4.4. Behavioral State Decoding and Neural-Feature Separability

From a decoding perspective, combining the power in the theta and alpha bands with network metrics allowed the models to extract information related to entrance-related behavioral states from multidimensional neural features. The classification performance was numerically highest for no-food-leave versus direct-leave, followed by direct-enter versus direct-leave, and it was lower for hesitation-leave versus direct-leave. This numerical ordering suggests that the NCL may exhibit a relatively distinctive overall activity pattern when pigeons look inside the maze arm and alter their initial approach behavior. The neural pattern associated with direct-enter was also distinguishable to some extent, whereas the activity patterns associated with hesitation-leave and direct-leave may have been more similar. Overall, decoding complemented the individual-metric analyses from a multivariate perspective. The decoding results indicated some neural-feature separability among entrance-related behaviors at the level of overall NCL activity patterns, beyond changes in individual power or network metrics.

### 4.5. Limitations and Future Directions

Several aspects of this study could be improved in future work. First, the study included only three pigeons and should therefore be regarded as a preliminary investigation. The limited number of independent animals restricts population-level generalization, and the present findings require confirmation in a larger independent cohort. The magnitude and statistical robustness of the neural differences also varied among pigeons, with P092 showing more pronounced state-related differences for several measures. Such interindividual variation may reflect natural differences in behavioral and neural responses, as well as small differences in recording location within the NCL. However, the present data do not allow these possibilities to be distinguished. Because the primary power, mean coherence, and functional network metric comparisons were conducted separately for each pigeon rather than by pooling trials across animals, the stronger effects observed in P092 did not influence the statistical outcomes for P106 or P653. Nevertheless, the observed interindividual variability indicates that the consistency of these patterns should be further evaluated in a larger independent cohort.

Second, behavioral states were classified based on pigeons’ observable behavior at the maze arm entrance. Although the occurrence frequency and overall behavioral response duration were quantified, fine-grained behavioral components, including visual sampling, within-response pauses, and head movements, were not analyzed in detail. Future work could combine eye tracking, head trajectories, and alignment to key behavioral events with more detailed temporal analyses. This approach could help to characterize dynamic NCL activity during approach, observation, and departure. In addition, the present study focused mainly on the theta and alpha bands in the NCL. Future studies could examine other frequency bands and record simultaneously from visual regions and the hippocampus. Analyses of interregional connectivity and cross-frequency coupling could provide a more complete understanding of the neural activity underlying information processing and behavioral adjustment at the maze arm entrance.

## 5. Conclusions

This study analyzed changes in NCL LFP activity and functional network features across behavioral states exhibited by pigeons at the maze arm entrance. Compared with direct-leave, the other three entrance-related behaviors were generally accompanied by higher power and interchannel coherence in the theta and alpha bands. The mean node strength and clustering coefficient also tended to be higher overall, whereas global efficiency and modularity exhibited more complex patterns. Behavioral state decoding based on power and functional network features further suggested that these neural features contained information relevant to differentiating the entrance-related behavioral states examined in this study. Overall, the findings indicate that NCL low-frequency neural population activity, interchannel coordination, and functional network organization are closely associated with entrance-related behavioral states in pigeons. These findings further suggest that the NCL may be involved in entrance-related information processing and subsequent behavioral adjustment. These preliminary findings provide new evidence regarding NCL neural activity associated with diverse entrance-related behavioral responses in pigeons. Future studies could integrate more detailed behavioral measurements with recordings from multiple brain regions to further characterize the associated neural dynamics.

## Figures and Tables

**Figure 1:**
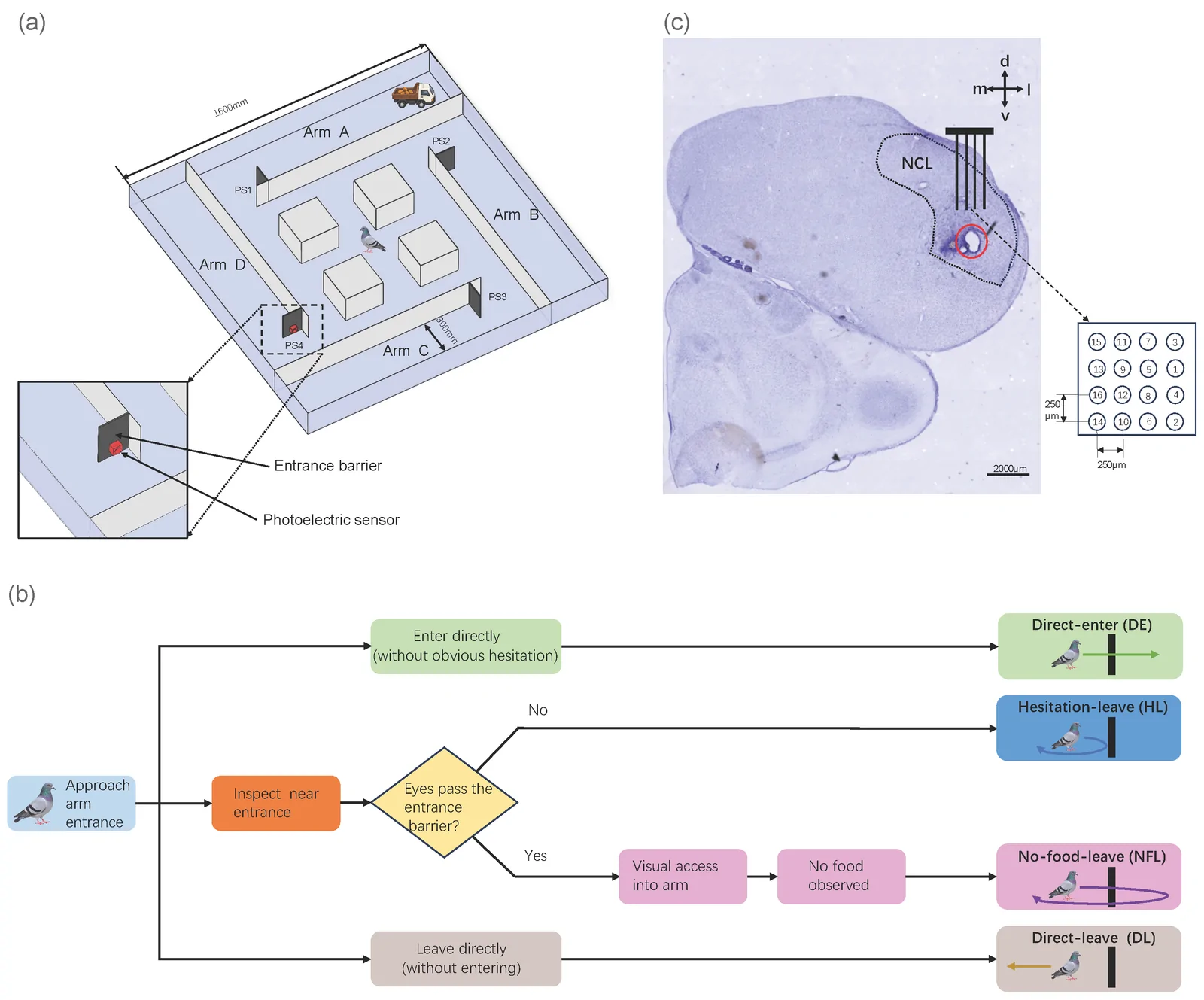
Maze apparatus, behavioral-state classification procedure, and electrode implantation site. (**a**) The square maze consisted of four arms (A–D), with photoelectric sensors (PS1–PS4) positioned at the entrance of each arm. The enlarged inset illustrates the entrance barrier and photoelectric sensor. (**b**) Sequential procedure used to classify entrance-related behaviors into direct-enter (DE), direct-leave (DL), hesitation-leave (HL), and no-food-leave (NFL) according to the predefined behavioral criteria. (**c**) Histological identification of the electrode implantation site. A representative Nissl-stained coronal section shows the approximate NCL boundary and the electrolytic lesion used to verify the recording site. The electrode array is shown schematically, and the inset indicates the channel numbering and spatial arrangement of the 16-channel microelectrode array.

**Figure 2:**
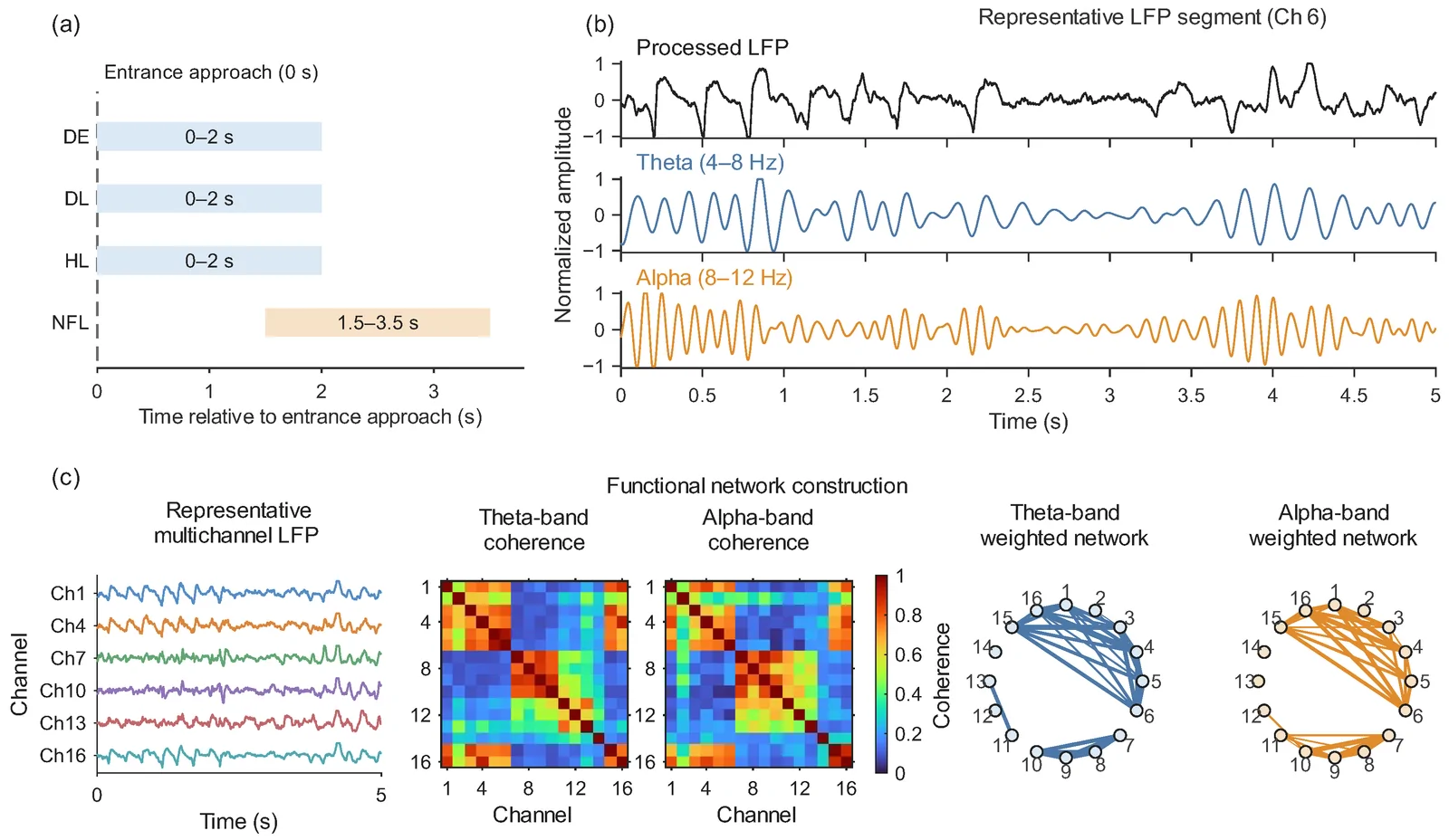
LFP preprocessing and functional network construction workflow. (**a**) Analysis windows used for the four entrance-related behavioral states. Direct-enter (DE), direct-leave (DL), and hesitation-leave (HL) were analyzed within the 0–2 s window, whereas no-food-leave (NFL) was analyzed within the 1.5–3.5 s window relative to entrance approach. (**b**) Representative LFP signal and its theta (4–8 Hz) and alpha (8–12 Hz) band components after preprocessing. (**c**) Schematic illustration of functional network construction. Representative multichannel LFP signals were used to calculate theta- and alpha-band coherence matrices, which were subsequently converted into weighted functional networks.

**Figure 3:**
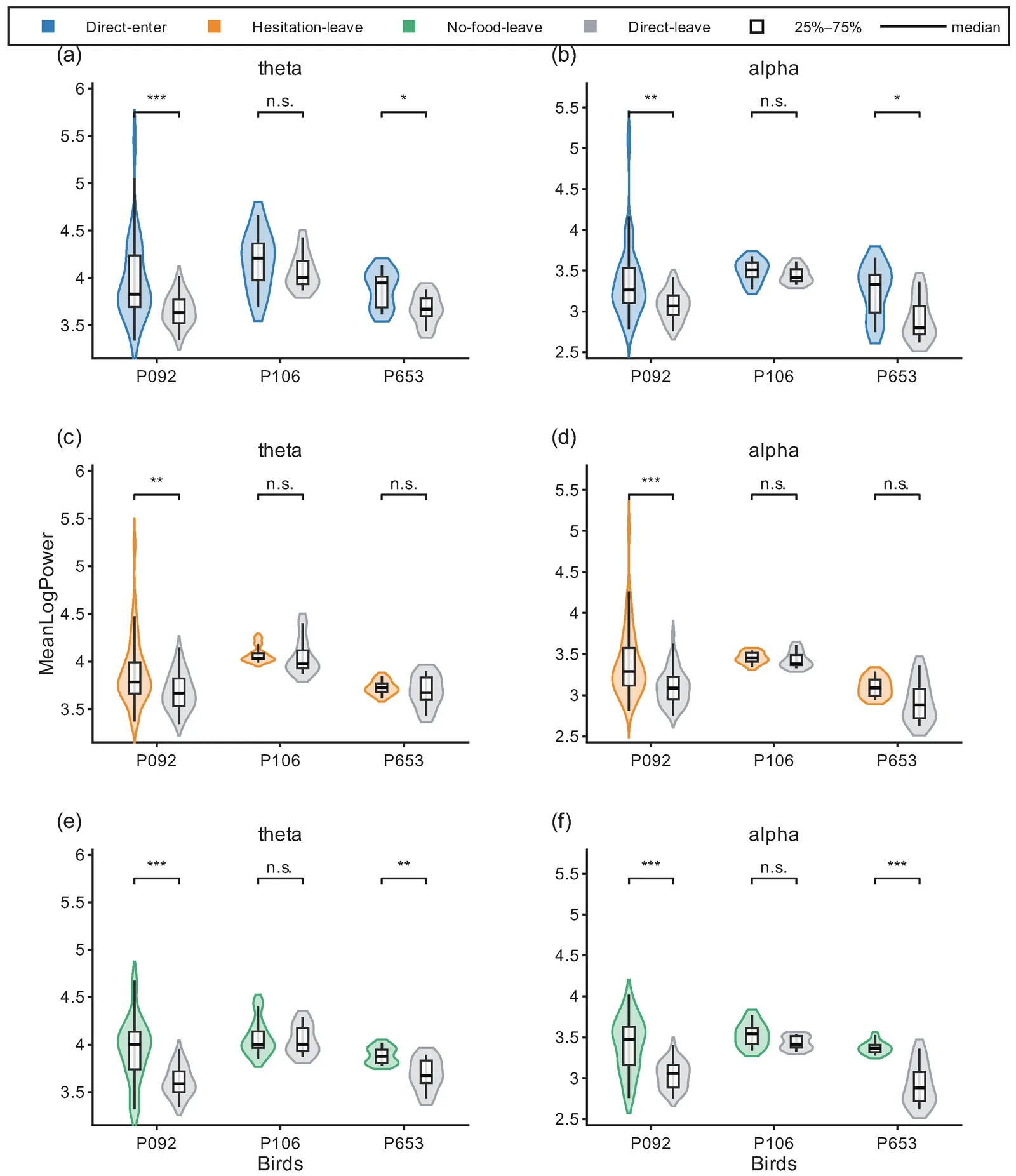
MeanLogPower distributions across entrance-related behavioral states in three pigeons. (**a**,**b**) Direct-enter versus direct-leave; (**c**,**d**) hesitation-leave versus direct-leave; (**e**,**f**) no-food-leave versus direct-leave. The left and right columns show the theta (4–8 Hz) and alpha (8–12 Hz) bands, respectively. *** p<0.001, ** p<0.01, * p<0.05, n.s. p≥0.05. Asterisks indicate raw *p* values; FDR-adjusted *q* values are reported in the main text.

**Figure 4:**
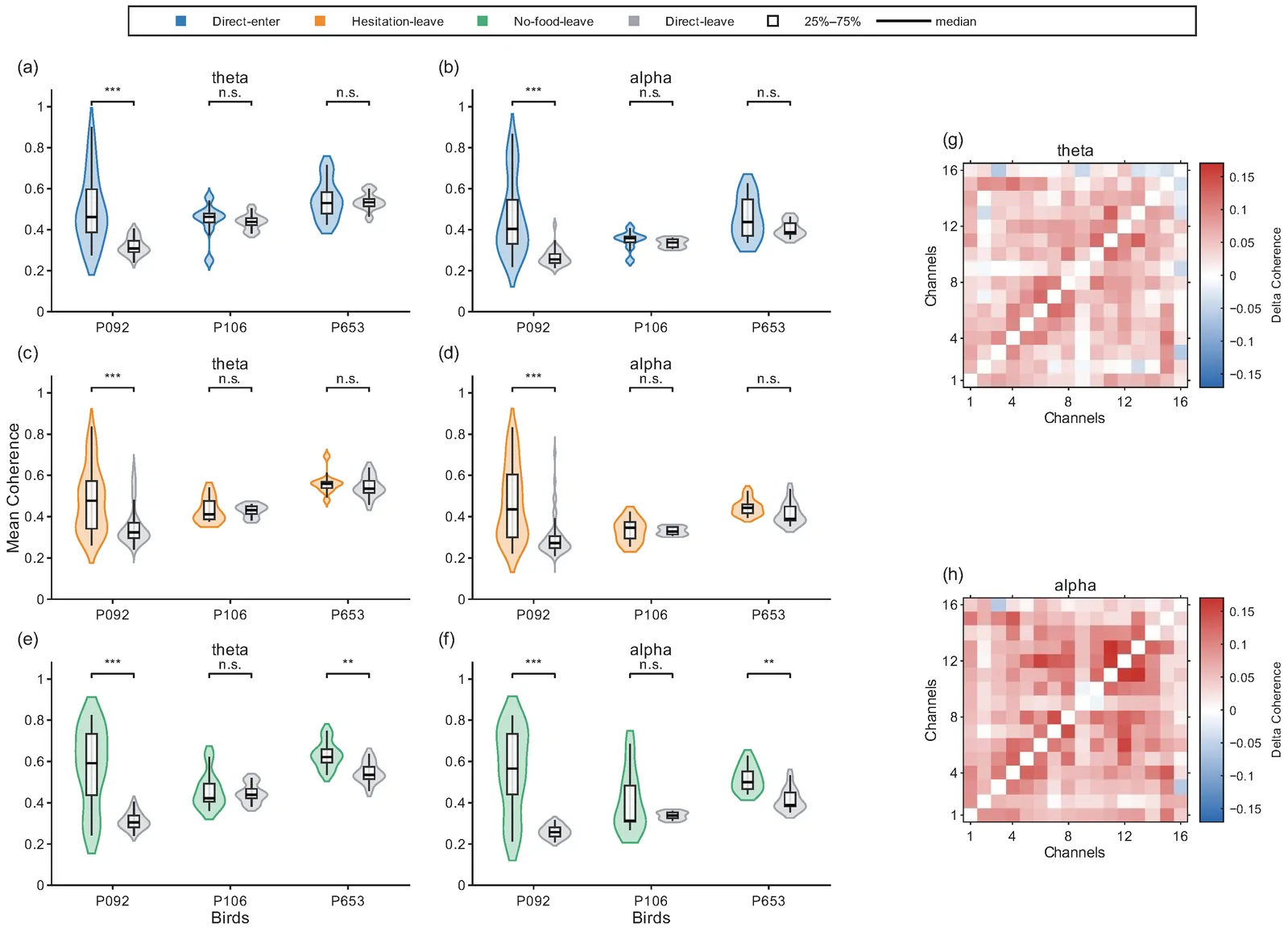
Mean coherence across entrance-related behavioral states in three pigeons. (**a**–**f**) Behavioral comparisons and frequency band arrangement are the same as in Figure 3. (**g**,**h**) Equally weighted mean coherence difference matrices across the three pigeons for hesitation-leave minus direct-leave in the theta and alpha bands, respectively. *** p<0.001, ** p<0.01, n.s. p≥0.05. Asterisks indicate raw *p* values; FDR-adjusted *q* values are reported in the main text.

**Figure 5:**
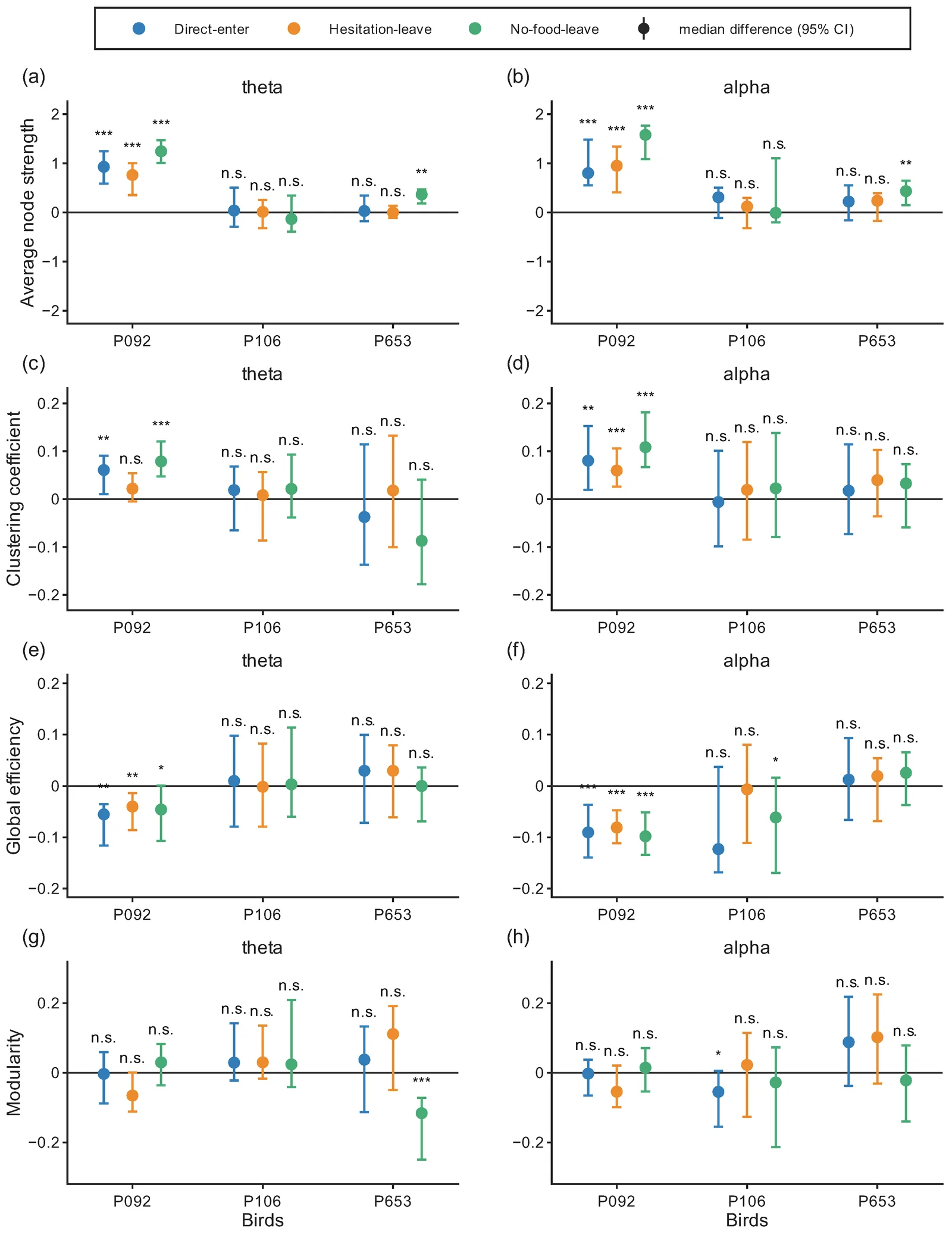
Functional network metrics across entrance-related behavioral states in three pigeons. (**a**,**b**) Mean node strength; (**c**,**d**) clustering coefficient; (**e**,**f**) global efficiency; (**g**,**h**) modularity. The left and right columns show the theta (4–8 Hz) and alpha (8–12 Hz) bands, respectively. Values represent the median difference between each behavioral state and direct-leave at a 25% network density. *** p<0.001, ** p<0.01, * p<0.05, n.s. p≥0.05. Asterisks indicate raw *p* values; FDR-adjusted *q* values are reported in the main text.

**Figure 6:**
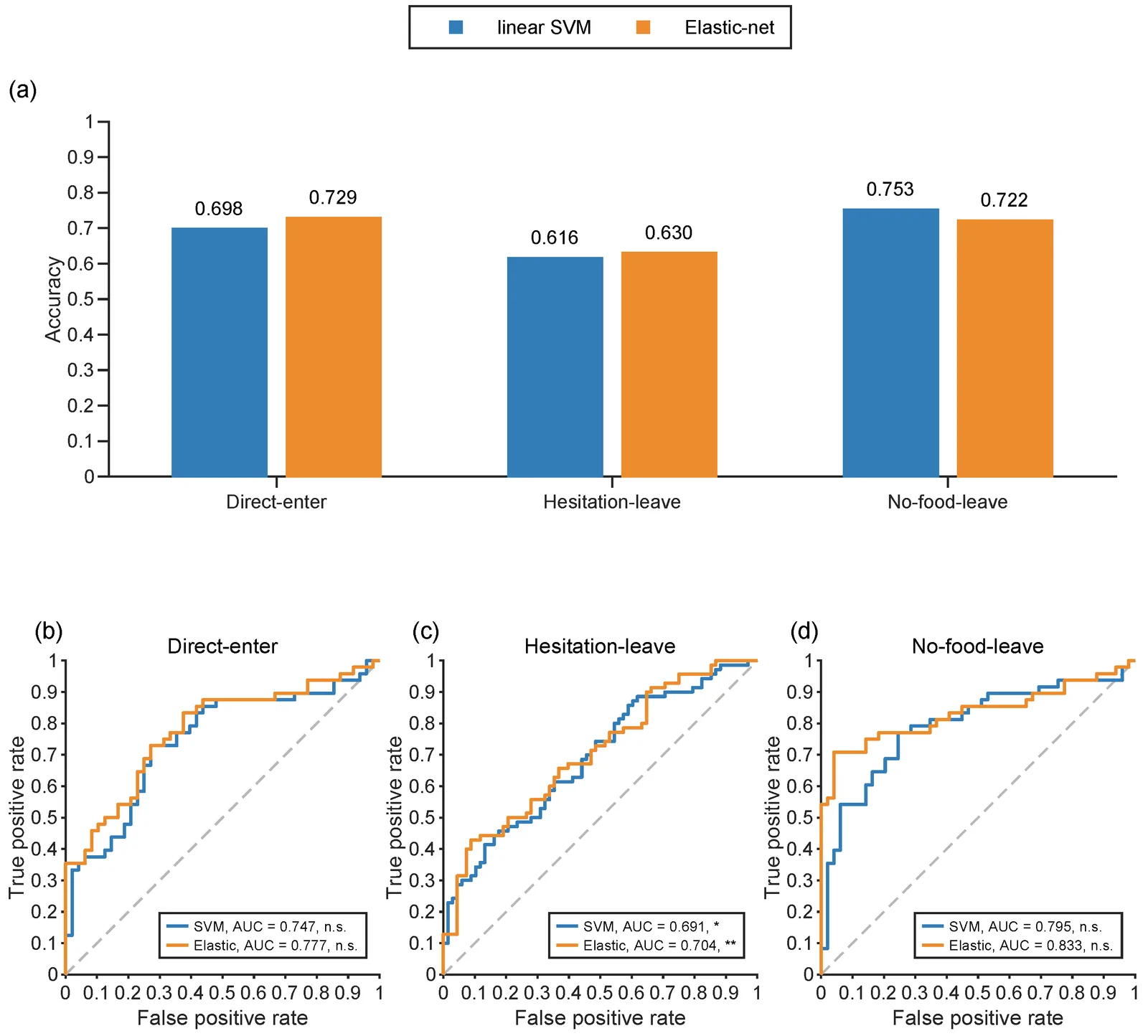
Leave-one-session-out decoding of entrance-related behavioral states based on power and functional network features. (**a**) Accuracy of the linear SVM and elastic-net logistic regression classifiers. (**b**–**d**) ROC curves for direct-enter versus direct-leave, hesitation-leave versus direct-leave, and no-food-leave versus direct-leave, respectively. The diagonal line indicates chance-level performance. Asterisks indicate raw *p* values from 500 within-session label permutations of AUC: ** p<0.01, * p<0.05, n.s. p≥0.05. FDR-adjusted *q* values are reported in the main text.

**Figure 7:**
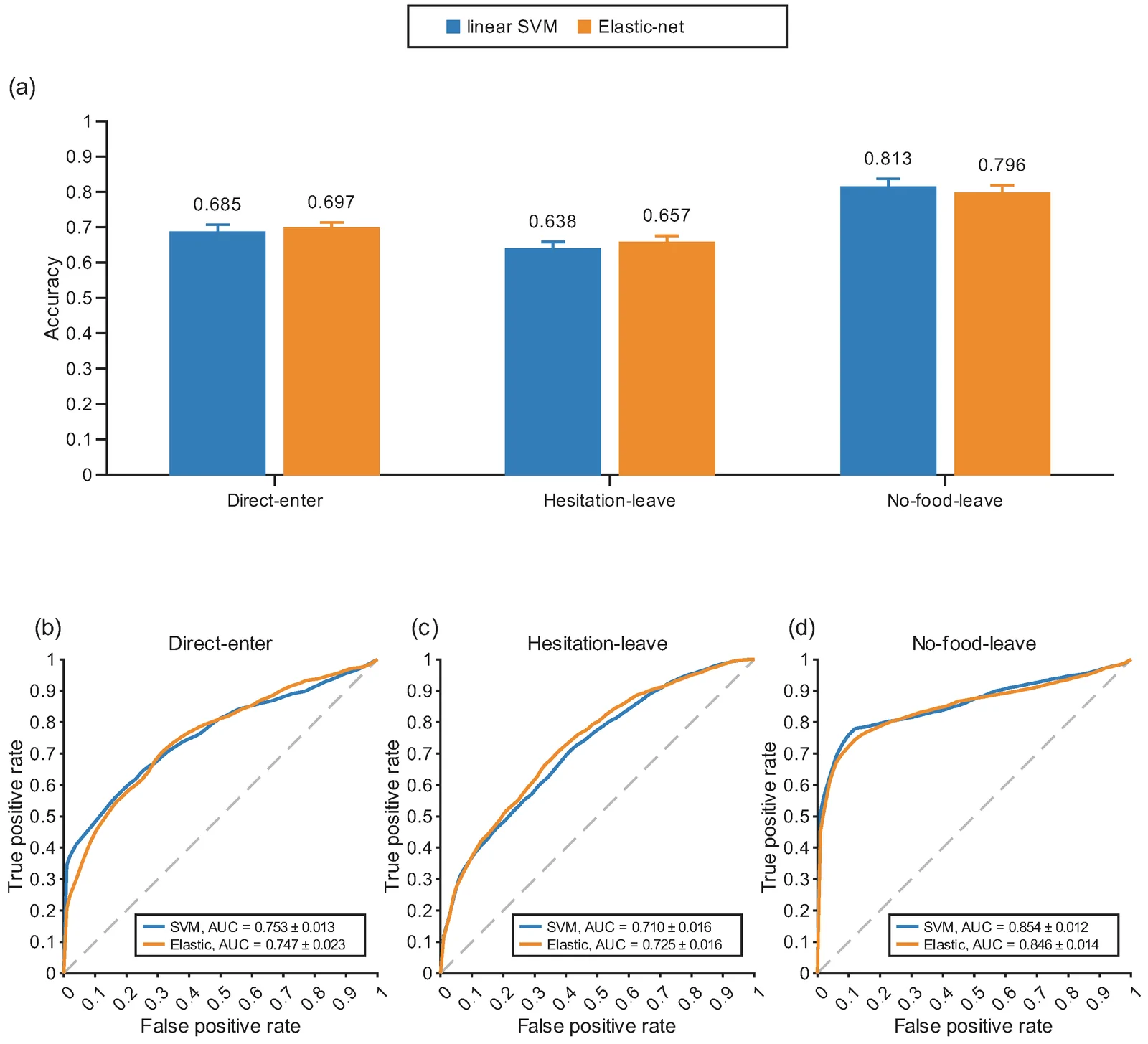
Repeated stratified 10-fold cross-validation decoding of entrance-related behavioral states based on power and functional network features. (**a**) Accuracy of the linear SVM and elastic-net logistic regression classifiers across 50 repetitions. (**b**–**d**) ROC curves for direct-enter versus direct-leave, hesitation-leave versus direct-leave, and no-food-leave versus direct-leave, respectively.

**Table 1:** Median (interquartile range) of MeanLogPower across behavioral states.

Behavioral Comparison	Band	Behavioral State	P092	P106	P653
Direct-enter vs. direct-leave	Theta	Direct-enter	3.829 (3.694–4.238)	4.210 (3.975–4.363)	3.947 (3.691–4.013)
		Direct-leave	3.588 (3.500–3.717)	4.004 (3.934–4.178)	3.674 (3.597–3.833)
	Alpha	Direct-enter	3.264 (3.106–3.530)	3.510 (3.422–3.599)	3.329 (2.987–3.451)
		Direct-leave	3.058 (2.886–3.162)	3.416 (3.373–3.518)	2.883 (2.721–3.075)
Hesitation-leave vs. direct-leave	Theta	Hesitation-leave	3.784 (3.664–3.989)	4.029 (4.025–4.088)	3.727 (3.682–3.769)
		Direct-leave	3.588 (3.500–3.717)	4.004 (3.934–4.178)	3.674 (3.597–3.833)
	Alpha	Hesitation-leave	3.289 (3.116–3.573)	3.454 (3.408–3.514)	3.090 (2.993–3.192)
		Direct-leave	3.058 (2.886–3.162)	3.416 (3.373–3.518)	2.883 (2.721–3.075)
No-food-leave vs. direct-leave	Theta	No-food-leave	4.002 (3.739–4.136)	4.003 (3.965–4.141)	3.876 (3.805–3.949)
		Direct-leave	3.588 (3.500–3.717)	4.004 (3.934–4.178)	3.674 (3.597–3.833)
	Alpha	No-food-leave	3.471 (3.159–3.629)	3.543 (3.418–3.610)	3.365 (3.319–3.410)
		Direct-leave	3.058 (2.886–3.162)	3.416 (3.373–3.518)	2.883 (2.721–3.075)

## Data Availability

The raw data supporting the conclusions of this article will be made available by the authors on request.
